# Disentangling response initiation difficulties from response inhibition in autism spectrum disorder: A sentence-completion task

**DOI:** 10.3389/fpsyg.2022.964200

**Published:** 2022-09-26

**Authors:** Joana C. Carmo, Carlos N. Filipe

**Affiliations:** ^1^CICPSI, Faculdade de Psicologia, Universidade de Lisboa, Lisbon, Portugal; ^2^NOVA Medical School, Universidade Nova de Lisboa, Lisbon, Portugal

**Keywords:** response initiation, response inhibition, autism spectrum disorder, Hayling test, sentence-completion task, executive system

## Abstract

It has been proposed that individuals with autism spectrum disorder (ASD) struggle both with response initiation and with response inhibition, both of which are functions of the executive system. Experimental tasks are unlikely pure measures of a single cognitive domain, and in this study we aim at understanding the contributions of response initiation difficulties to possible deficits in inhibitory control in autism. A sample of adults diagnosed with ASD and a control sample participated in this study. To participants it was asked to perform a sentence-completion task with two different condition: Part A—targeting response initiation and Part B—engaging inhibitory processes. Importantly, we have analyzed the B-A latencies that have been proposed for the removal of the response initiation confound effect. Results show that no differences between the groups were found in accuracy measures, either in Part A (ASD: *M* = 0.78; Controls: *M* = 0.90) nor Part B (ASD: *M* = 0.03; Controls: *M* = 0.02). However, in both conditions autistic participants were significantly slower to respond than the group of participants with typical development (Part A—ASD: *M* = 2432.5 ms; Controls *M* = 1078.5 ms; Part B—ASD *M* = 6758.3 ms; Controls *M* = 3283.9 ms). Critically, we show that when subtracting the response times of Part A from Part B (B-A latencies) no group differences attributable to inhibitory processes remained (ASD: *M* = 4325.76; Controls: *M* = 2205.46). With this study we corroborate the existence of difficulties with response initiation in autism and we question the existence of troubles in inhibition *per se.*

## Introduction

For the fact that a dysfunction of the executive system in Autism Spectrum Disorder (ASD) is one of the main accounts for the primary symptoms of this condition (Russel, 1997) a considerable large amount of research have been devoted over the last decades to the study of the executive system functioning in ASD. This proposal might be best in explaining a core diagnostic feature in ASD—the presence of repetitive behaviors and restricted interests (Hill, 2004). Autism, is a developmental disorder marked by the presence of at least one core symptom in three different domains: social interaction; communication and repetitive behaviors and restricted activities (see for instance Wadhera and Kakkar, 2019). The recent edition of Diagnostic and Statistical Manual of Mental Disorder (DSM—5th edition) have combined different diagnostic labels (e.g., Asperger syndrome or Autism disorder) under a single umbrella term “ASD” stressing the idea of a spectrum of deficits on the different domains (Wadhera and Kakkar, 2019).

The executive system, at use for adaptive responses to novel or complex situations, encompasses many distinct and diverse functions from planning and working memory to inhibition and mental flexibility but also initiation of a response, all of which are primarily attributed to the frontal lobes (Stuss, 1992). Yet it is accepted now that autistic individuals might not encounter problems across all executive domains (Geurts et al., 2004). Importantly, however, is the fact that the majority of tasks designed to measure executive functions depend on non-executive functions, as perception or memory, and also problematic is the fact that each task is never a pure measure of a single executive domain (Pennington and Ozonoff, 1996; Geurts et al., 2004).

Several studies addressed and have evaluated the pattern of preserved vs. spared functioning of distinct executive domains in ASD. However, the picture is not simple and not always consistent. One of the seminal works by Ozonoff and Jensen (1999) evaluated both cognitive flexibility and planning (with the Tower of Hanoi) and found these domains to be impaired in their sample. The same sample of autistic children show, however, a preservation of inhibition of a response (in the Stoop test) (Ozonoff and Jensen, 1999). Another multi-task study that also included the Stroop test and the Tower of Hanoi, reported preserved functioning of inhibition and planning, respectively, and also preserved functioning of cognitive flexibility, with only the working memory domain being compromised (Goldberg et al., 2005). A different approach is used in other studies where, for a given executive domain more than one task was considered. In Geurts et al. (2004) several tasks were applied aiming to evaluate the contributions to planning, working memory, flexibility, fluency and inhibition and found that all but the working memory component were compromised. In the same line, another study included a battery of seven different tasks aiming at evaluating their contribution to planning/working memory, flexibility and response inhibition. In this study, with children and adolescents, it was reported, from their composite scores, a poor performance of the ASD sample on inhibition and planning/working memory but not on cognitive flexibility (Happé et al., 2006). In this study, it was also compared the different profiles in young vs. old participants and results show that the differences observed were only present for the younger group. Authors concluded that this age-related improvements are indicative that executive functioning deficits may ameliorate with increasing age (Happé et al., 2006). A study focused on eye movement responses evaluated different executive domains from infancy to adulthood as they aimed to assess delayed or impaired development (Luna et al., 2007). They evaluated response initiation, response inhibition and working memory components, and found, overall, troubles in all domains. The autistic participants were slower in the latency to initiate memory guided saccades, but this finding was only present in adult participants. In response inhibition and working memory there was overall a poor performance, but both the ASD and control samples showed developmental improvements, yet with differences in the stabilization/adult level response (Luna et al., 2007). A 10-year longitudinal study evaluated the trajectories of executive functions from childhood into adulthood, with a 2- and 10-years follow ups (Fossum et al., 2021). This study focused on working memory component, inhibition (Color-Word interference test) and on planning abilities, and revealed that at baseline (12 years old in average) the ASD sample had a poorer performance than the control sample, on all domains. Importantly, they found that although the performance gap was not reduced along time, the maturation slopes were similar across groups, which led the authors to concluded that their results support the hypothesis of a similar rather than diverging developmental trajectories (Fossum et al., 2021).

The diversity and complexity of tasks and the different approaches used on studying executive functioning in ASD makes it difficult for a clear or overarching account, however, at least two review studies concur on the existence of deficits in both cognitive flexibility and planning (Pennington and Ozonoff, 1996; Hill, 2004). A recent review study concurs also on the existence of deficits in cognitive flexibility (but not on planning) and found also support for deficits on the working memory component (St. John et al., 2022), which were also reported in the Pennington and Ozonoff (1996) review. Regarding inhibitory control, these review studies reported either that findings were rather inconclusive (St. John et al., 2022) or that inhibition of a response in this population was unimpaired for neutral responses or inhibitory deficit *per se*, but that autistic individuals might be moderately impaired, or impaired in certain cases, in inhibiting prepotent responses (Pennington and Ozonoff, 1996; Hill, 2004).

Several studies have been devoted to the study of inhibitory processes in ASD and it has been acknowledged the importance of using multiple measures to evaluate a single cognitive construct (Christ et al., 2007). Inhibition is a component of the executive system that allows individuals to withhold prepotent responses or ignore distracting stimuli (Adams and Jarrold, 2012), for which individuals must suppress the activation or processing of information that would otherwise interfere with the achievement of a cognitive or behavioral goal (Dempster, 1992). The main tasks used to assess inhibitory control are the Stroop test, the Go/no-go task and also the Flankers task, where the firsts are considered measures of prepotent response inhibition and whereas the latter is a task of resistance to distractor interference (Adams and Jarrold, 2012).

In ASD it has been proposed that individuals might encounter troubles in some aspects of the inhibitory control but not all. Specifically, it has been proposed that autistic individuals have a selective deficit with an impaired ability when needing to ignore distracting information but preserved inhibition of prepotent responses (Christ et al., 2011). In accordance, it has been shown in quite a few studies that the performance of ASD children is unimpaired in Stroop tasks (Ozonoff and Jensen, 1999; Goldberg et al., 2005; Christ et al., 2007). Also in a tasks analogous to the Stroop test (e.g., the D-KEFS word interference task) it was reported that both in children and adults the performance is preserved (Christ et al., 2011; Mostert-Kerckhoffs et al., 2015, but see Corbett et al., 2009 for contrasting results in children). Along the same line, it has been generally found a preserved performance in a Go/no go task (or in an analogous Stop-signal task), where the performance of children seems to be preserved (Ozonoff and Strayer, 1997; Happé et al., 2006; Christ et al., 2007; Adams and Jarrold, 2012), which, however, contrasts with recent findings of Wilson et al. (2014) in adults, and in children by Schmitt et al. (2019). Regarding the Flankers task, the pattern is the opposite with the majority of studies reporting that ASD children have considerable difficulties with this type of tasks (Christ et al., 2007, 2011; Adams and Jarrold, 2012, but see Ozonoff and Strayer (1997) that reported preserved performance in children in a distractor task). Notwithstanding, results of a meta-analysis regarding inhibitory control in ASD show that both prepotent response inhibition and resistance to distractor interference were found to be deficient in ASD individuals, and strikingly with a larger effect for measures of prepotent inhibition which seems to be more difficult for ASD individuals than interference control (Geurts et al., 2014). Moreover, in a meta-analysis of imaging studies it was found, for adult participants, a difference in convergence in the right inferior frontal gyrus when contrasting with control samples, when performing inhibitory tasks. When comparing autistic adults with a younger sample a divergence of convergence on the left inferior frontal gyris was observed. These findings indicate that these areas are likely to be recruited to solve inhibition tasks in autistic adults, but that abnormalities are not evident until adulthood (Zhang et al., 2020).

For studying response inhibition another task, a sentence-completion one, has been used. The Hayling test constructed by Burgess and Shallice (1996) allows the direct comparison between response inhibition and response initiation, with minimal changes across conditions. The idea that both these processes are subserved by similar and overlapping functional systems have been tested and demonstrated (Burgess and Shallice, 1996; Nathaniel-Jones et al., 1997). Importantly is the fact that, in any inhibition tasks, such as the Stroop test, the inability to suppress the most salient response is related to the ability or not to initiate a response (see Burgess and Shallice, 1996). In the Hayling test, sentences are presented to the participant with the last word omitted (e.g., “Most sharks attack very close to the ________”). All sentences, randomly assigned to either Part A—response initiation or Part B—response inhibition, have a very high probability of a particular response. In Part A, in the one hand, participants are asked to provide an ending that makes sense (obvious response) and that fits the sentence context. On the other hand, in Part B participants must provide an ending word which makes no sense in the context of the sentence, hence the high-probability ending word must first be suppressed (Burgess and Shallice, 1996). Error scores and latencies can be analyzed. These authors propose also a score in which the latencies of Part A are subtracted from Part B (B-A latencies) in order to remove the confound factor of initiation problems when considering the latencies in the response suppression condition (Burgess and Shallice, 1996).

A handful of studies have used the Hayling test in Autism, both in adults (Boucher et al., 2005; Hill and Bird, 2006; Johnson et al., 2011) and in children (Robinson et al., 2009), reporting conflicting results. If in the one hand, the Haying test performance in autistic children seem to lead to significant differences, both in error scores and in response times in the inhibition condition but not in part A (Robinson et al., 2009). Also, in the seminal work of Boucher et al. (2005) on the Hayling test with adolescents and adults, in which only error score were reported, differences for the inhibition—Part B condition were observed, with an increased number of errors for the ASD sample as compared to the control group. On the other hand, the work of Hill and Bird (2006) and Johnson et al. (2011) showed no error score differences for neither Part A nor Part B. These two studies, with adult participants, did, however, analyzed the response latencies of both conditions and found overall that the autistic participants took longer than their control samples when performing both Part A and Part B. Neither of these two studies have reported the B-A latencies differences.

In contrast to response inhibition, or other executive functions, the initiation of a response in ASD is likely the less studied executive domain, despite of its importance to a host of behaviors. Two recent reports based on first-hand accounts from people on the spectrum have stressed the importance of this executive function on their condition perception. Welch et al. (2019) study gives some hints regarding the perceived mismatch between what is seen on the outside (i.e., behaviors; e.g., not making eye contact) and what is going on in the inside (i.e., intentions; e.g., interest in social contact) (Welch et al., 2019). Along the same line, Buckle et al. (2021) studied inertia—the inability in acting on the intentions—with focus groups and first-hand accounts from adults with ASD. An overarching theme found in this study was the difficulty in initiating tasks of any type, even simple ones. Scaffolding to support action was also a consistent theme found, with prompting from the external environment leading to promoting actions in this population (Buckle et al., 2021). In an experimental setting, Carmo et al. (2015, 2017) have analyzed the performance in a verbal fluency task as function of time in adults with ASD, and observed that in the first time period this group of participants produced fewer words as compared to the control group but that in the remaining time periods the differences between groups were no longer present. These authors considered that this pattern of results could signal a deficit at the initiation of the task, and have also showed that with a simple external verbal cue the deficit was no longer observable (Carmo et al., 2017).

With the current study we aim at further investigating both initiation and inhibition processes in ASD, in a single sentence-completion task with minimal changes across conditions and interchangeable stimuli (adapted from the Hayling test and extended). High-functioning adult participants diagnosed with ASD and an age-, gender-, schooling- and general cognitive abilities- matched control sample performed this sentence-completion task, with two conditions: response initiation (Part A) and response inhibition (Part B). Critically, we evaluated in ASD the contribution of possible initiation difficulties to the overall inhibition (Part B) response times by analyzing the B-A latencies, as proposed by Burgess and Shallice (1996). As mentioned before, each task is never a pure measure of single component of the executive system, and initiation of a response is particularly pervasive to a multitude of functions and behaviors, and have been considerably neglected. If it is the case that initiation of a response processes are impaired in this population, we anticipate that when correcting for this confound effect, the putative inhibitory difficulties in ASD might no longer be observable.

## Methods

### Participants

A total 32 participants took part in the experiment. Eighteen neurotypical participants and 14 participants with a diagnosis of ASD composed the control and ASD group, respectively. ASD participants were selected if: (i) They were more than 18 years old; (ii) they had more than 9 years of formal education and (iii) they scored above 80 points in the verbal subscale of the Wechsler Adult Intelligence Scale (WAIS). The ASD diagnosis was carried out by an experienced psychiatrist and was based on the DSM-V (American Psychiatric Association, 2013). The diagnosis of ASD was further confirmed by the Autism Diagnostic Observation Schedule (ADOS) (Lord et al., 1999) and/or by the Asperger’s Syndrome Diagnostic Scale (ASDS) (Myles et al., 2001). The two samples were matched for age, schooling and general cognitive abilities as measured by the RAVEN colored matrices (see Table 1). Written informed consent was obtained from each participant prior to any experimental procedure, and the study was approved by the ethical committee of the first author’s Faculty (in accordance with the Declaration of Helsinki).

**TABLE 1:** Participant’s demographic information.

	ASD	Controls	*P*-value
*n*	14 (1 Female)	18 (1 Female)	
Age	26.43 (5.99) [19–43]	27.61 (4.70) [21–43]	0.54
Schooling (years)	14.00 (1.84) [12–17]	14.56 (1.82) [12–17]	0.40
RAVEN (raw scores)	52.00 (4.67) [40–59]	53.17 (3.11) [47–58]	0.40

### Material and procedure

A sentence-completion task was constructed by adapting and extending the Hayling test (Burgess and Shallice, 1996). The Hayling test consists of 30 sentences, divided by two conditions (Part A, Part B), in which the final word is omitted (e.g., “To keep the dogs out of the yard he put up a _______”) that were selected from Bloom and Fischler (1980). For our study, 50 sentences from the Bloom and Fischler (1980) norms for sentence completion were selected if scored high in response probability, and two set of 25 randomly assigned sentences each were created (see Bloom and Fischler, 1980, for more details). The two sets did not differ in the probability scores (Set 1: *M* = 0.92, *SD* = 0.043; Set 2: *M* = 0.92, *SD* = 0.046; *p* = 0.80). The sentences were translated and audio recordings were produced. Each of the two sets were assigned to either Part A (Initiation) or Part B (Inhibition) thus two versions of the task were built.

Participants performed always Part A before Part B, in a fixed order and the trial structure was kept the same across conditions and only the written instructions changed. In the Part A—Initiation—participants were instructed to listen to each sentence in which the last word was omitted and were asked to try to provide a word that would fit at the end of the sentence. In Part B—Inhibition—participants also listen to the sentences with the last word omitted, but were asked instead to try to provide a word that would make no sense in the context of the sentence. Four training trials were given in each of the two conditions, and the experiment began if participants had understood the task correctly.

Each trial started with a fixation cross slide and this inter-trial interval varied from 500 ms to 1000 ms, randomly assigned. Following, participants listened to one sentence with headphones and were asked to press a button as soon as possible if already knew the answer. Reaction Times were collected at this point. Immediately after, participants were asked to write down the sentence completion word. The 2 versions of the task built were counterbalanced across participants, and each participants completed 25 randomized trials in each condition. All participants were tested individually in a quiet room and the experiment was run through e-prime 2.

## Results

### Accuracy measures

For the fact that in the Initiation section of the task (Part A) there were no wrong answers we proceed to evaluate values of entropy, as a measure of response variability. Here, the modal responses were analyzed by calculating each trial’s entropy. *Entropy* = – Σ *pi log2 (pi)*, where *pi* is the proportion of a given response among total answers (as in Magyari et al., 2014, but see Shannon, 1948). When entropy values are close to zero (low entropy) most participants give the same verbal response and the values of high entropy are the cases in which most responses were different. A pair sample *t*-test was run with the results of entropy of each sentence for the Control and ASD samples, and no differences were observed [*t*(49) = 1.117, *p* = 0.269] and very low scores (close to zero) were obtain for either group (see Table 2).

**TABLE 2:** Accuracy measures for both Part A and Part B sections of the sentence completion task.

	Controls	ASD	*P*-value
Part A—InitiationEntropy	0.90 (0.10)	0.78 (0.11)	0.269
Part B—InhibitionAccuracy	0.20 (0.05)	0.30 (0.08)	0.170
Part B—Strategies %			
Context (C)	8.91 (2.49)	4.74 (1.34)	0.183
Last answer (L)	6.90 (2.05)	4.74 (1.48)	0.424
C + L	2.86 (1.43)	0.93 (0.93)	0.297

Mean results by group are presented (and Standard Error from the mean).

For the Inhibition section (part B) responses were given zero points for correctly completed sentences (unrelated response), one point for a semantically related word, and three points for an incorrect completion (obvious responses) (as in Burgess and Shallice, 1996; Robinson et al., 2009). An independent *t*-test show that no differences were observed between the two groups [*t*(30) = 1.405, *p* = 0.170] (see Table 2). Strategy use was analyzed following the procedure by Burgess and Shallice (1996) where correct answers were classified in two main different strategies used. Responses could be classified as a Context (C) response if they would correspond to objects normally present in a lab/office (e.g., computer, keyboard) or Last answer (L) if their response was semantically related to the last word given. On the occasions where both condition were satisfied the response was considered C + L. Independent *t*-tests were run on these three categories and results show that no differences in strategy use between the groups were observable (all *p*s > 0.18) (see Table 2).

### Reaction times

**FIGURE 1:**
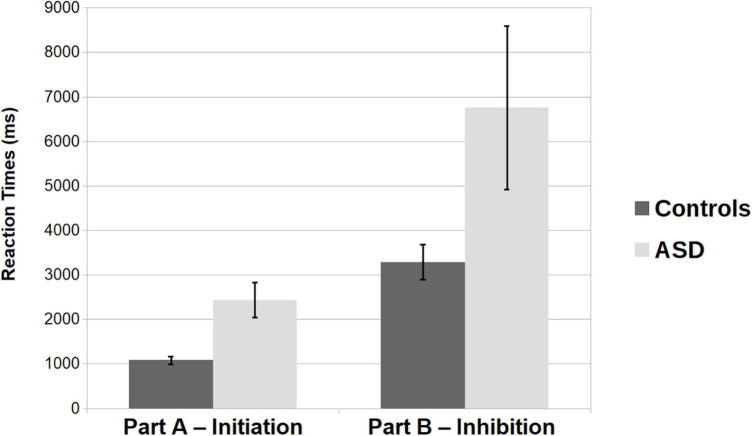
Average reaction times (in ms) for the Control and the ASD groups (light gray) both for Part A and Part B sections of the sentence completion task. Error bars depict the standard deviation from the mean.

Reaction times were analyzed for correct responses. In part B we considered incorrect responses the obvious response (to be avoided) and its synonyms and antonyms. Outlier responses that fell out of 100 ms < RTs > 3*SDs interval were excluded from the analysis. An ANOVA with Condition (Initiation, Inhibition) as a within-subjects variable and with Group (Controls, ASD) as a between-subjects factor was run (see Figure 1). Results show a main effect of Condition [*F*(1, 30) = 28.927; *p* < 0.001; η*_*p*_*^2^ = 0.491] with responses in Part B (Inhibition) taking in average a longer time (*M* = 5021.12, *SEM* = 837.41) than in Part A (*M* = 1755.51, *SEM* = 260.43). The main effect of Group [*F*(1, 30) = 4.984, *p* = 0.033; η*_*p*_*^2^ = 0.142] was found to be significant as well, with the ASD sample being in average slower (*M* = 4595.43, *SEM* = 811.08) than the control group (*M* = 2181.21, *SEM* = 715.31). The interaction effect was found not significant (*p* = 0.091).

As in Burgess and Shallice (1996) we analyze, for each subject, the latency differences of Part B minus Part A section (B-A latencies). An independent *t*-test shows that the differences between the groups, when removing the initiation confound, were no longer significant [*t*(30) = –1.746, *p* = 0.091; Controls: *M* = 2205.46, *SEM* = 376.07; ASD: *M* = 4325.76, SEM = 1294.75]. Please see Table 3 for a comparison with relevant literature.

**TABLE 3:** Results’ summary for studies that have employed the Hayling test in autism spectrum disorder.

Study	Group (sample size)	Age group (mean age)	Dependent measures			
			Part A—Initiation		Part B—Inhibition	
			Error scores	Response times	Error scores	Response times
Robinson et al. (2009)	ASD (54)Controls (54)	Children ASD (150. 46 months) Controls (144.96 months)	ns	ns	ASD *M* = 5.41; Controls *M* = 3.41 (*F*) = 5.07*	ASD *M* = 3.13; Controls *M* = 2.33 (*F*) = 5.56*
Boucher et al. (2005)	ASD (10)Controls (10)	Adolescents and Adults ASD (23 years) Controls (24 years)	NR	NR	ASD *M* = 14.5; Controls *M* = 3 (*F*) = 8.094*	NR
Hill and Bird (2006)	Asperger (22)Controls (22)	Adults Asperger (32.09 years) Controls (33.45 years)	ns	ASD *M* = 22.86; Controls *M* = 6.91*	ns	ASD *M* = 60.57; Controls *M* = 25.23*
Johnson et al. (2011)	ASD (24)Controls (14)	Adults ASD (27.8 years) Controls (28.7 years)	NR	ASD *M* = 20.4; Controls *M* = 4.4 (*F)* = 7.73**	ns	ASD *M* = 52.3; Controls *M* = 17.8 (*F*) = 4.17*
Current study	ASD (14)Controls (18)	Adults ASD (26.4 years) Controls (27.6 years)	ns	ASD *M* = 2432.5; Controls *M* = 1078.5 *t* = 0.14*	ns	ASD *M* = 6758.3; Controls *M* = 3283.9 *t* = 0.047*

* for p-value < 0.05 and ** for p-value < 0.01; NR for results not reported and ns for non-significant results.

## Discussion

In our study we aimed at disentangling the contribution of initiation processes difficulties in autism from putative difficulties in inhibitory processes in this population. Adult participants with high-functioning autism and a well-matched control sample performed a sentence-completion task, with minimal changes across conditions and interchangeable stimuli, similar to the Hayling test (see Burgess and Shallice, 1996). This task comprised two different conditions, presented in a fixed order, in which Part A engaged processes regarding the initiation of a response, whereas in Part B, participants were required to inhibit a prepotent response (Burgess and Shallice, 1996). Critically we analyzed the B-A latencies—subtraction of participants’ response times of Part A from the response times of part B—as proposed by Burgess and Shallice (1996) for the evaluation of the impact of response initiation difficulties, which have been reported in ASD, on response inhibition abilities.

Foremost, our results on the performance of Part A show that already in this task condition participants with ASD were considerably slower than their control group (ASD: *M* = 2432.5; Controls *M* = 1078.5). This result is in line with the results obtain in a verbal fluency task, where differences in performance were observed in participants with ASD in the first time bin analyzed, whereas in the remaining of the task the differences between them and the control group were no longer present (Carmo et al., 2015, 2017). These results were interpreted by the authors as indicative of troubles at the initiation of a response in autism, which is corroborated by the present findings. The hypothesis of an impairment in response initiation in ASD has received recent reinforcement by first-hand accounts from individuals with ASD, in which a common theme disclosed was the difficulty in initiating tasks of any type (Buckle et al., 2021). Critically to this issue, is the fact that external environmental prompting have been reported, either experimentally or from first-hand accounts, to ameliorate initiation difficulties in this population (Carmo et al., 2017; Buckle et al., 2021), which is of great importance to change implementations in practice. This prompting aid could, for instance, be easily implemented in professional settings, where a supervisor could provide some external cues in the beginning of each task, and hence facilitate the difficulties in task initiation. Besides this external cues aiding, the fact that the latencies in initiation of a response increase the overall time of execution, it could be implemented, for instance in educational settings, an adjustment in terms of the amount of time allowed to complete a given task (e.g., extra time for an exam). Considering that the initiation of a response is needed for possibly all kind of overt behaviors, this line of research is quite auspicious and deserving of attention in future studies, ultimately seeking changes in applied settings that could significantly impact the daily lives of those with this condition.

Results regarding performance scores on both task conditions (entropy and accuracy measures) show no observable differences between the two groups (*p*s > 0.17) with very low entropy and very low amount of errors, respectively, and are as such, in line with the results of both Hill and Bird (2006) and Johnson et al. (2011), disconfirming as well the results reported by Boucher et al. (2005) in their seminal work with the Hayling test in adolescents and adults with ASD (see Table 3 for a better comparison with relevant literature).

At a first glance our results on response times would indicate that adults with ASD indeed struggle with inhibitory processes (ASD *M* = 6758.3 ms; Controls *M* = 3283.9 ms), as it have been proposed in the literature (e.g., Geurts et al., 2014). Our findings on reaction times are also consistent with findings of the Hayling test on adult population (see Table 3). Even if in our sample, participants with ASD were perfectly able to suppress the prepotent response and were able provide a correct response, with comparable strategy use, they might have needed additional processing time to do so. Yet when considering the B-A latencies results, we show that the differences observed for part B (Inhibition condition) were no longer observed (ASD: *M* = 4325.76 ms; Controls: *M* = 2205.46 ms). In this analysis, proposed by Burgess and Shallice (1996) in order to eliminate the confound effect of response initiation difficulties, we subtracted from the average response times in Part B, the average response times in the initiation condition (Part A), and our results indicate that indeed no differences between the ASD and control groups might be attributable to inhibition processes, but rather to difficulties in the initiation of a response. This result is quite important and questions the deficits reported not only in studies with the Hayling tests (e.g., Hill and Bird, 2006; Johnson et al., 2011), but with all sorts of tasks targeting inhibitory processes in autism, in particular tasks requiring the suppression of a prepotent response (e.g., Wilson et al., 2014; Schmitt et al., 2019, for Go/no Go tasks). Highlighting the importance of taking into account this somehow neglected executive function when evaluating cognitive hypothesis in ASD, for its pervasive impact on virtually all overt behaviors and cognitive domains. Regarding, particularly, studies of inhibitory processes in autism (but not exclusively), a component of the executive function that have been proposed to be impaired in this population, it would be strongly recommended to design future studies that are able to account for or to remove the impact of response initiation delays in ASD, which have now received some additional empirical support.

The current study evaluated initiation of a response and inhibition abilities of adult participants exclusively, and we advise against any generalization to younger autistic population. Results on age and development regarding executive functions in autism, namely on inhibitory processes, are quite discrepant, with impairment being observed in children only, ameliorating with increasing age (Happé et al., 2006), or present both in children and adult samples (Fossum et al., 2021), or that brain abnormalities are observable in adults only (Zhang et al., 2020). Likely developmental differences, and the time course of maturation of the frontal lobes, are the reasons for what we consider generalizations are to be avoided when studying any executive function. Regarding processes of response initiation our results would be consistent with existent studies on this executive function, that reported, for instance, troubles on initiation in adult samples only (Luna et al., 2007), or children’s preserved performance on the Hayling test (see Table 3). Note, however, that this does not mean that generalization should be carried out, and that only with further developmental studies on response initiation we will be able to understand whether initiation impairments are really only observable later in development.

At last, some limitations of the current work, and a summary of main recommendations should be mention. In the first place, our sample size is relatively small which makes, particularly, the results on the accuracy measures, which were found non-significant, of deserving further evaluation with a considerably larger sample, and to be taken with cautious. Secondly, we have tested a sample of adults participants only, for what results should not be generalized to other age groups. We found this developmental aspect of particular interest given the results on the Hayling test reported for younger samples. As already mention, it would be important to understand whether impairments reported regarding response initiation in autism are really only present or observable in adult populations or if they appear early in development.

As further recommendation we would like to add that general results on inhibitory control in ASD, or on other executive domains, might be impacted by response initiation deficits and further direct evaluation would be critical. Moreover, our study does not address the potential beneficial impact of external cues or prompting on response initiation. The fact that this mitigation of response initiation difficulties is of great importance and could impact the daily life of autistic individuals, it should definitely deserve further and pressing attention.

In sum, we have shown that when removing initiation of a response delays in participants with ASD, differences regarding inhibitory processes are no longer observed, questioning the reported deficits of this executive function in autism. We offer, as well, additional support for the hypothesis of an impairment of processes of response initiation in autism, a function that is likely involved in a multitude of behaviors and cognitive domains and that should deserve cautious and careful attention when designing future studies on autism. The importance of studying the initiation of a response also reflects the fact these deficits that have been shown to ameliorate by external prompting, which could lead to changes in practical setting, with a considerable impact on the lives of those with autism.

## Data availability statement

The data that support the findings of this study are available from the corresponding author, upon request.

## Ethics statement

The studies involving human participants were reviewed and approved by the Comissão de Ética e Deontologia da Faculdade de Psicologia da Universidade de Lisboa. The patients/participants provided their written informed consent to participate in this study.

## Author contributions

JC and CF contributed equally to the design of the study and interpretation. JC collected and analyzed the data and draft the manuscript. CF revised the manuscript. Both authors contributed to the article and approved the submitted version.
